# Role of cholecystectomy in hyperkinetic biliary dyskinesia: A systematic review and meta-analysis

**DOI:** 10.1016/j.sopen.2025.06.001

**Published:** 2025-06-03

**Authors:** Duyen Quach, Kayla Nguyen, Gabriella Tavera, Rachel Wright, Zuhair Ali, Mike Liang

**Affiliations:** a Department of Surgery, Graduate Medical Education, HCA Healthcare, Kingwood, TX, United States of America; b Tilman J. Fertitta Family College of Medicine, University of Houston, Houston, TX, United States of America

**Keywords:** Hyperkinetic gallbladder, Hyperkinetic biliary dyskinesia, Biliary hyperkinesia, Cholecystectomy

## Abstract

**Background:**

Biliary dyskinesia is disorder characterized by reduced gallbladder ejection fraction, which have shown a good response to cholecystectomy. In contrast, hyperkinetic biliary dyskinesia (HBD), as defined by ejection fraction ≥80 %, is an emerging phenomenon, and the role of cholecystectomy is not yet clearly defined. This review investigates the effectiveness of cholecystectomy in alleviating symptoms of HBD.

**Material and methods:**

A comprehensive literature search was conducted to retrieve studies based on predefined inclusion criteria. Data were extracted by two-independent reviewers. A random-effects model was used for meta-analysis. Risk ratios (RR) were calculated to estimate the impact of cholecystectomy on symptom improvement. Heterogeneity was calculated using the I^2^ statistic and Q-test, with subgroup analyses performed based on study design.

**Results:**

Fourteen studies involving 416 patients with HBD were included. Overall, the pooled RR for symptom relief post-cholecystectomy was 3.72 (95 % CI: 2.57–5.38). A subgroup analysis of retrospective reviews showed an RR of 3.9 (95 % CI: 2.57–5.92). Moderate heterogeneity (I^2^ = 30.01 %) was observed.

**Conclusion:**

Based on existing evidence, cholecystectomy appeared to be a promising and effective treatment for HBD in select patients.

## Introduction

Hyperkinetic gallbladder, also known as hyperkinetic biliary dyskinesia (HBD) is a condition marked by excessive gallbladder contractions, usually defined as a gallbladder ejection fraction of 80 % or more on cholecystokinin-hepatobiliary iminodiacetic acid (CCK-HIDA) scintigraphy. [1] Patients with HBD often experience symptoms similar to that of gallbladder disease, such as abdominal pain, nausea, and vomiting. Despite its clinical significance, HBD remains largely underrecognized in clinical practice due to variability in diagnostic criteria and limited awareness among clinicians [2].

In evaluating biliary symptoms, abdominal ultrasound is typically the first diagnostic step, but more specialized imaging such as hepatobiliary iminodiacetic acid (HIDA) is infrequently used to assess for biliary obstruction when abdominal ultrasounds are unequivocal. Even less commonly performed is the CCK-HIDA scan, which measures gallbladder contractility and is usually performed to evaluate functional gallbladder disorder. This modality, though underutilized, has allowed HBD to gain recognition as a potential cause of abdominal symptoms in patients with otherwise normal imaging findings. As diagnostic technologies advance, HBD is now gaining recognition as a possible cause of abdominal symptoms, even in patients who do not show typical signs of gallbladder disease [3]. Accurately diagnosing and effectively managing this condition are increasing important for clinicians, particularly in the field of general surgery, as it impacts a large portion of the patient population.

Although advances in diagnostics have led to increased recognition of HBD, gaps remain in the literature regarding its diagnosis and management. The use of CCK -HIDA is inconsistent in clinical practice, partly due to a lack of consensus on interpretation thresholds. For example, an EF of 80 % is commonly used as a marker of gallbladder hyperkinesis, but Ziessman et al. suggested that this value overlaps with normal physiology and emphasizes the need for standardized diagnostic criteria [4]. While some studies suggest cholecystectomy may help relieve symptoms in patients with HBD, the overall evidence is limited, and no clear consensus exists [5,6]. Attributing HBD as a cause of abdominal pain is further complicated by the finding that some patients undergoing cholecystectomy for HBD were found to have evidence of cholecystitis on final pathology [[7], [8], [9]]. This raises the question of whether cholecystitis was the primary cause of their symptoms, or if it developed as a result of the underlying hyperkinetic condition.

To address existing knowledge gaps and provide clearer clinical guidance, a comprehensive systematic review and meta-analysis are warranted. This review hypothesizes that cholecystectomy, when compared to non-operative management, could lead to complete symptom relief in patients with HBD. By systematically gathering and analyzing available evidence from relevant studies, this review will evaluate the effectiveness of cholecystectomy for resolving symptoms in patients with HBD. Beyond assessing outcomes, this review aims to also clarify diagnostic criteria and treatment strategies to identify and manage HBD more effectively. Ultimately, this review seeks to offer evidence-based recommendations for the diagnosis and management of HBD in clinical practice.

## Methods

### Study design

This systematic review adhered to the Preferred Reporting Items for Systematic Reviews and Meta-Analyses (PRISMA) guidelines. A comprehensive literature search was conducted to identify relevant studies examining the role of cholecystectomy in patients diagnosed with hyperkinetic gallbladder (ejection fraction ≥80 %), compared to non-operative management, in resolving biliary colic symptoms and improving quality of life.

### Search strategy

The search strategy was developed to retrieve studies from electronic databases including PubMed, Embase, and Cochrane Library. The search was conducted using a combination of Medical Subject Headings (MeSH) terms and keywords related to hyperkinetic gallbladder, cholecystectomy, and outcomes of interest. The search covered studies from inception to May of 2024.

The search strategy included the following terms:

(biliary hyperkinesia OR gallbladder hyperkinesia OR hyperkinetic gallbladder OR hyperkinetic biliary dyskinesia OR gallbladder with high ejection fraction) AND (cholecystectomy OR removal of the gallbladder)

### Study selection

Following the initial search, duplicate records were removed. Two independent reviewers (DQ and GT) screened the titles and abstracts of studies based on predefined inclusion criteria. Full-text articles were then retrieved and reviewed for eligibility. Any discrepancies in study selection were resolved through consultation with a third reviewer.

Inclusion criteria were defined using the PICO format:

• Population: Patients with gallbladder ejection fraction equal or >80 %
• Intervention: Cholecystectomy
• Comparison: Non-operative management
• Outcomes: Symptom resolution, improvement in quality of life measures, adverse events following cholecystectomy

Exclusion criteria:

• Studies conducted on pediatric populations or animals
• Studies involving adults with gallbladder EF <80 %
• Studies not specifically addressing hyperkinetic gallbladder or evaluating cholecystectomy as a treatment intervention
• Studies with inadequate data or insufficient detail to assess outcomes related to cholecystectomy

### Data extraction

Data from each included study were extracted by two independent reviewers (DQ and GT) using a standardized data extraction form. Information gathered included:

• Study characteristics (author, year, study design)
• Patient demographics (age, sex, gallbladder ejection fraction)
• Details on the intervention (cholecystectomy)
• Outcomes (symptom resolution, adverse events, improvement in quality of life).

### Quality assessment

The quality of included studies was evaluated based on study design. Case reports and case series were assessed using the Joanna Briggs Institute (JBI) Critical Appraisal Checklist. This tool evaluates aspects such as clarity in reporting of patient demographics, intervention details, and outcome measurement. Retrospective reviews were evaluated using the Newcastle-Ottawa Scale for cohort studies, which assesses the selection of participants, comparability of study groups, and the measurement of outcomes [10]. Two independent reviewers performed the quality assessments. Discrepancies were resolved through discussion, or by consulting a third reviewer if needed. Studies were rated as having low, moderate or high risk of bias based on the evaluation criteria.

### Data synthesis and statistical analysis

A narrative synthesis was conducted to summarize the characteristics and outcomes of the included studies, focusing on symptom improvement, symptom resolution and adverse events following cholecystectomy. Given the observational nature of the studies, a meta-analysis was performed to pool the effect sizes across studies to provide estimates for future randomized controlled trial development. The analysis incorporated retrospective reviews, case series, and case report, each of which was analyzed separately in subgroup analysis to explore the influence of study design on outcomes.

To address potential variability between studies, a random-effects model (using the Restricted Maximum Likelihood (REML) approach) was employed. This model assumes that the true effect size may vary across studies due to differences in populations, methodologies, and study designs, providing a more conservative estimate of the pooled effect size.

### Risk ratio (RR) calculation

The risk ratio (RR) was used to quantify the effect of cholecystectomy on symptom improvement. The RR was calculated as the ratio of the probability of symptom improvement based on the available data (e.g., the number of patients with symptom improvement versus those without improvement). The pooled RR was calculated using a random-effects model to accommodate potential variability between studies. The results are presented with 95 % confidence intervals (CIs) to provide a range of plausible effect sizes. An RR >1 indicates a higher likelihood of symptom improvement following cholecystectomy.

### Assessment of heterogeneity

Heterogeneity across the included studies was assessed using the I^2^ statistic and the Q-test. The I^2^ statistic measures the percentage of total variation across studies that is due to heterogeneity rather than chance. The I^2^ statistic can roughly be interpreted as [11]:

• 0–40 %: Negligible heterogeneity
• 30–60 %: Moderate heterogeneity
• 50–90 %: Substantial heterogeneity
• 75–100 %: Considerable heterogeneity

The Q-test evaluates whether the differences in effect sizes across studies exceed what would be expected by chance, with a *p*-value <0.10 indicating significant heterogeneity.

### Subgroup analysis

A subgroup analysis was performed based on study design to assess how much of the overall data was skewed by case reports and case series, which typically only report positive outcomes of symptom improvement following cholecystectomy. Since these studies do not provide data on patients who did not improve, their inclusion may disproportionately influence the pooled effect size. By analyzing only the retrospective reviews, we aimed to compare these results back to the overall analysis, allowing for a clearer understanding of the true effect of cholecystectomy on symptom improvement in patients with biliary hyperkinesia.

### Protocol registration

This systematic review was registered with PROSPERO (registration # CRD42024590821) and can be accessed at https://www.crd.york.ac.uk/prospero/display_record.php?RecordID=590821.

## Results

### Study selection

**Fig. 1:**
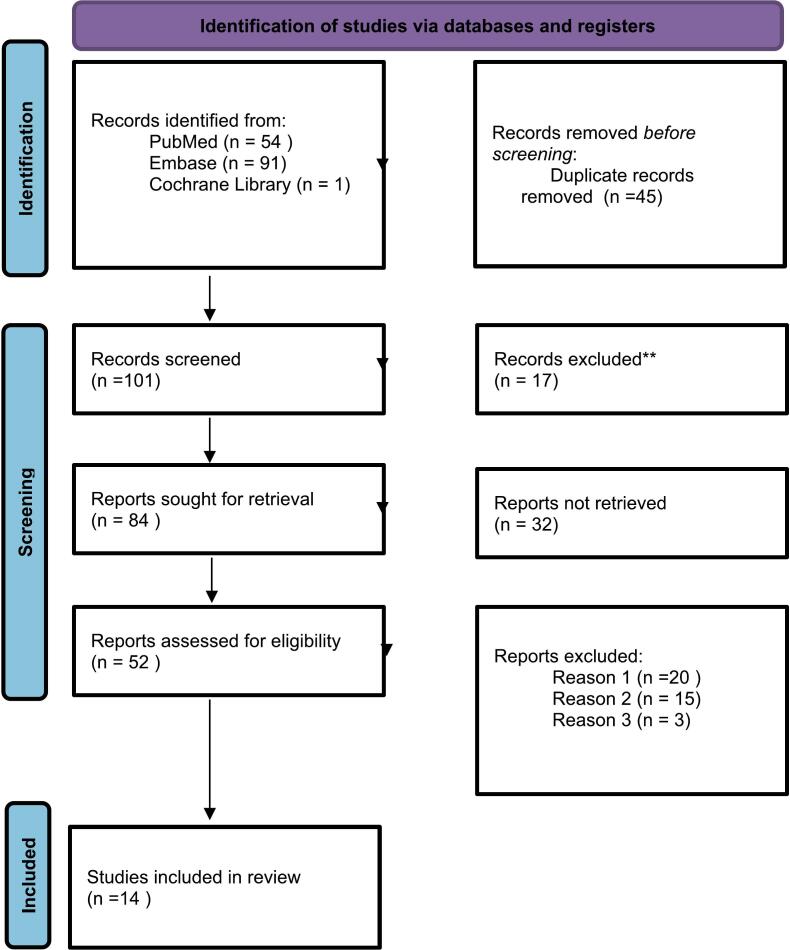
PRISMA flowchart. **Reports excluded due to (1) not meeting the study inclusion criteria (2) irrelevance to the research question (3) insufficient data.

Our systematic search yielded a total of 146 studies. After removing 45 duplicates, 101 studies were screened based on titles and abstracts. Following further evaluation, 17 studies were excluded for not meeting the inclusion criteria, resulting in 84 reports sought for retrieval. Ultimately, 14 studies were included in the final review after excluding full text articles due to (1) not meeting the inclusion criteria, (2) irrelevance to the research question and/or (3) insufficient data. The PRISMA flowchart illustrating the study selection process is presented in Fig. 1.

### Study characteristics

The included studies consisted of retrospective reviews (*n* = 9), case series (*n* = 4), and case reports (*n* = 1) with sample sizes ranging from 1 to 101 participants. The median patient age was 41.2 years (range: 24.5 and 55.5 years), with gallbladder ejection fractions consistently ≥80 %. A summary of key study characteristics is provided in Table 1.

**Table 1:** Demographics, findings, and results of Included Studies.

Study	Year	Design	N	Avg age (years)	Ejection fraction (%)	Symptom improvement
Steele et al	2014	CS	2	24.5	92.5	2
Alexida et al	2017	RR	17	46	>75	15
Saurabh et al	2018	RR	31	NR	92	28
Sadek et al	2018	CS	2	55.5	>80	2
Gazzetta et al	2019	RR	77	40.5	90.9	70
Nasri et al	2019	RR	59	48.8	88.5	45
Pallapothu	2019	RR	41	NR	>80	40
Williford et al	2020	RR	18	NR	>80	16
Falco et al	2020	CS	3	40.3	96.1	3
Singh et al	2021	CS	3	36.3	91.3	3
Chu et al	2021	RR	13	43.9	>80	10
Black et al	2021	CR	1	33	99	1
Hart et al	2022	RR	48	41.2	87.3	46
Kartik et al	2023	RR	101	48	>81	79

CR = Case Report; CS = Case Series, RR = Retrospective Review, NR = not reported.

### Symptom improvement across studies

Among the 14 studies included in this systematic review, a majority reported symptom improvement in patients who underwent cholecystectomy, with varying rates of improvement across studies. Symptom improvement was reported in over 70 % of patients following surgery in some studies. For example, Kartik et al. reported a 79 % symptom improvement rate among 101 patients undergoing cholecystectomy, while Gazetta et al. demonstrated a 70 % improvement rate in 77 patients [8,12]. Outcomes from case reports and case series also showed favorable results, with all patients experiencing symptom improvement following cholecystectomy. In the case series by Falco et al., all 3 patients exhibited symptom resolution post-surgery [3]. These findings indicate that cholecystectomy may offer some benefit, though most studies did not include statistical comparisons to non-operative management.

Notably, only one study, Williford et al., included data on non-operative management, comparing symptom improvement between patients who underwent cholecystectomy and those who were managed conservatively [5]. Among 68 patients with EF >80 %, 18 patients underwent cholecystectomy and 50 patients received non-operative management. While this study suggested that patients who underwent cholecystectomy reported greater symptom improvement than those managed non-operatively, it is important to note that the majority of studies did not assess or report outcomes for non-operative management. As a result, the impact of non-operative strategies on symptom resolution remains unclear in the broader literature.

### Overall effect size and forest plot analysis

**Fig. 2:**
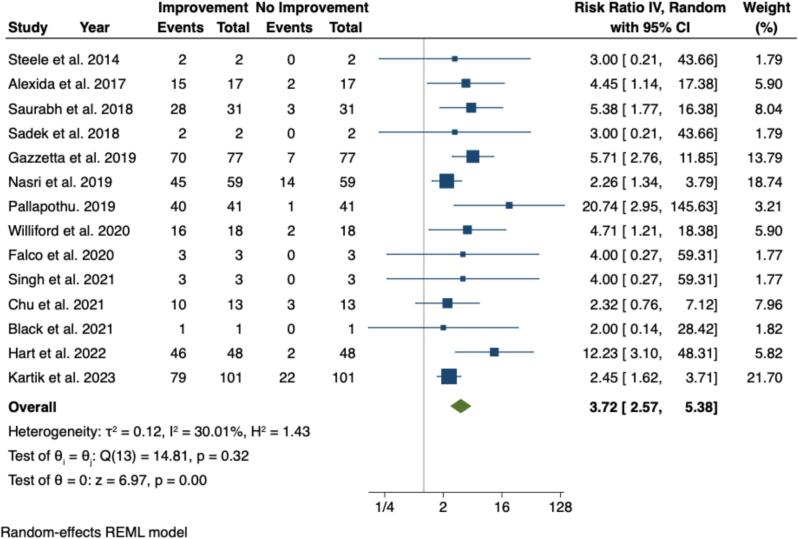
Forest plot showing the overall effect of cholecystectomy on biliary hyperkinesia.

The forest plot (Fig. 2) presents a pooled effect size from the 14 studies included in this review. The pooled analysis yielded a risk ratio (RR) of 3.72 (95 % CI: 2.57–5.38), indicating a significant association between cholecystectomy and symptom improvement. This suggests that patients with hyperkinetic gallbladder (EF >80 %) were 3.7 times more likely to experience symptom relief post-cholecystectomy compared to those who did not undergo the procedure.

The heterogeneity across studies was moderate with I^2^ = 30.01 % and H^2^ = 1.43, suggesting some variability in study outcomes. The τ^2^ value of 0.12 indicates small between-study variance. The Q-test for heterogeneity (Q(13) = 14.81, *p* = 0.32) suggests that much of the variability could be explained by chance. A random-effects REML model was applied to account for differences between studies, and the pooled effect was statistically significant (*p* < 0.001).

### Subgroup analysis by study design

**Fig. 3:**
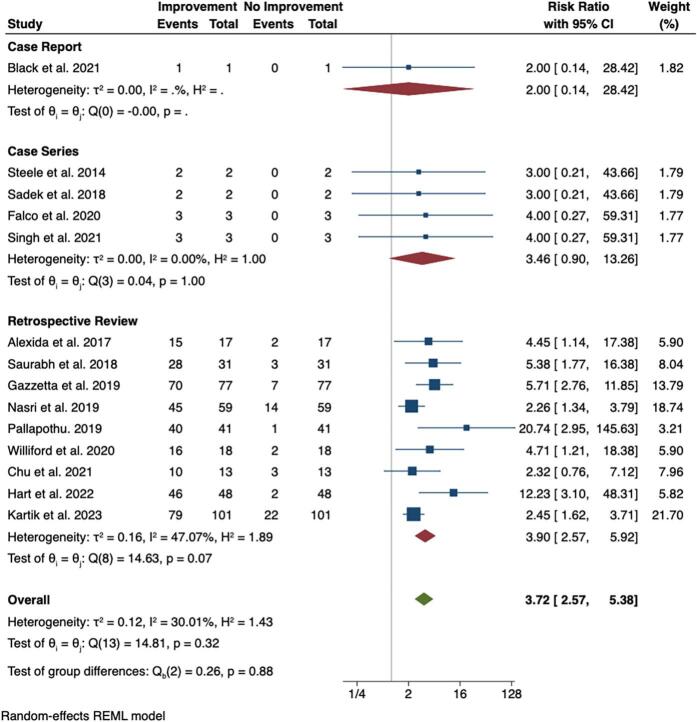
Forest plot with subgroup analysis showing effect of cholecystectomy on biliary hyperkinesia.

To explore the effect of study design on the outcomes, a subgroup analysis was performed, categorizing the studies into case reports, case series and retrospective reviews. The results are presented in Fig. 3.

Case Report: Only one case report, by Black et al. reported a risk ratio of 2 (95 % CI: 0.14–28.42) [13]. However, the wide confidence interval reflects uncertainty due to the small sample size, with only one event reported. This study contributed 1.82 % of the overall weight, with no heterogeneity within the group, as expected with a single case report.

Case Series: Four studies were included in the case series subgroup, yielding a pooled RR of 3.46 (95 % CI: 0.90–6.31) [3,6,14,15]. Although the effect size was favorable, the wide confidence interval suggests some uncertainty, possibly due to the limited sample sizes. There was no heterogeneity (I^2^ = 0 %, H^2^ = 100 %) within this subgroup. Indicating consistent results across studies. Each study contributed 1.77–1.79 % of the overall weight.

Retrospective Reviews: The retrospective reviews subgroup included 9 studies, which accounted for the majority of the overall weight in the meta-analysis [1,5,8,12,[16], [17], [18], [19], [20]]. The pooled RR was 3.90 (95 % CI: 2.57–5.92), showing a statistically significant benefit from cholecystectomy. The heterogeneity within this group was moderate (I^2^ = 47.07 %, τ^2^ = 0.16), likely due to variability in study populations, follow-up durations, and sample sizes. Kartik et al. contributed 21.70 % of the total weight, with an RR of 2.45 (95 % CI: 1.62–3.71), reflecting the influence of larger studies [8]. Notably, Hart et al. reported an exceptionally high RR of 12.23 (95 % CI: 3.10–48.31) [16]. Though the wide confidence interval highlights uncertainty due to smaller sample size.

### Heterogeneity and subgroup differences

The heterogeneity across the studies was assessed using the I^2^ statistic. In this review, the I^2^ value was 30.01 %, indicating moderate heterogeneity across the studies included in the meta-analysis. This heterogeneity could be attributed to differences in study design, patient populations, and diagnostic criteria for hyperkinetic gallbladder across the various studies. The test for subgroup differences yielded Q(2) = 0.26, *p* = 0.88, indicating that the effect size was consistent across study designs. This suggests that the benefits of cholecystectomy are observable regardless of the type of study design (case reports, case series, or retrospective reviews).

## Discussion

Our systematic review provides valuable insights into the role of cholecystectomy in managing patients with hyperkinetic biliary dyskinesia (HBD). While cholecystectomy is widely accepted as the treatment of choice for patients with biliary dyskinesia as defined as an ejection fraction below 35 %, its effectiveness in cases of hyperkinetic biliary dyskinesia with EF >80 % is not well established [2]. To our knowledge, only one previous systematic review, published in 2020, has evaluated the role of cholecystectomy for HBD, suggesting a potential benefit but the evidence was limited by the lack of control groups in the included studies [21]. Through this systematic review and meta-analysis, we evaluated the growing body of evidence supporting hyperkinetic dyskinesia as a surgical indication while highlighting critical gaps that limit the strength of conclusions.

To conduct a sound systematic review and meta-analysis, our approach included a rigorous search methodology, clear selection criteria, and thorough assessment of the included studies using the JBI Critical Appraisal Checklist for case reports and series, and the Newcastle-Ottawa Scale for retrospective reviews [10,22]. Despite these efforts, several limitations inherent to the current evidence base remain. Similar to prior review, our analysis was also limited by the quality of available data and its retrospective nature. The existing studies consist of retrospective reviews, case reports, and case series alone. This increased the risk of bias or methodological flaws. Additionally, our review may be limited by the inherent variability in study designs and patient populations, which could affect the generalizability of our findings. Finally, publication bias remains a potential drawback, as studies with positive outcomes are more likely to be published, which could skew the overall results of our meta-analysis. Addressing these biases through more comprehensive reporting in future studies will be helpful in clarifying surgical vs. medical management of HBD.

The quality and heterogeneity of available data presented significant challenges. Definitions of biliary pain and functional gallbladder disorder varied widely across studies, and most relied on subjective measures of symptoms relief, such as asking patients if they felt better after surgery. Objective measures of symptom relief, including validated quality-of-life indices like SF-36 or the GI Quality of Life Index, were rarely used. The reliance on subjective assessments introduces potential bias, which could influence the perceived efficacy of cholecystectomy.

The absence of a universally accepted gold standard for diagnosis limits the evaluation of HBD. Definitions of HBD, thresholds for abnormal gallbladder ejection fraction, and characterization of biliary symptoms varied widely across studies, resulting in considerable diagnostic heterogeneity. Imaging protocols, especially with regard to CCK-HIDA scans, were applied inconsistently across studies, with little reporting on infusion techniques, CCK dosing, and ejection fraction calculation, making standardization and comparison difficult.

Standardized infusion protocols for CCK-HIDA scans were proposed in 2010, recommending the use of sincalide at a dose of 0.02 μg/kg infused over 30 to 60 min to simulate physiologic conditions [23]. However, most of the studies included in our review did not specify whether such protocols were followed, raising concern about the accuracy of EF values reported in earlier literature. Rapid CCK infusion methods have been associated with falsely elevated or depressed ejection fractions, raising concerns about potential overdiagnosis or underdiagnosis of gallbladder dysfunction. As an alternative to synthetic CCK, some have proposed that fatty meal ingestion, such as a high-fat drink like Ensure Plus, may better replicate natural physiologic gallbladder activity during HIDA scanning in place of synthetic CCK [24]. While potentially more reflective of real-world gallbladder function, the fatty meal test also lacks standardization in terms of caloric content, and imaging timing and patient preparation, limiting its clinical utility. Evidence supporting its use remains sparse, and variability across institutions further complicates its widespread adoption.

Looking ahead, several questions remain regarding the optimal diagnostic pathway for HBD. Currently, synthetic CCK remains the standard agent used during HIDA scans, but variation in dosing and infusion protocols, or lack thereof in published studies, weakens the consistency and comparability of results across institutions. Standardization of CCK infusion rates and interpretation criteria will improve the reliability of HIDA scan results. Given the high cost of HIDA scans, which range from $700 to $1200 in the United States, variations in protocol are particularly concerning from both a diagnostic and healthcare resource perspective.

The choice of 80 % as the threshold for hyperkinetic biliary dyskinesia also warrants further scrutiny. As highlighted by prior research, including Ziessman et al., a gallbladder ejection fraction of 80 % or higher may overlap with normal physiological values, depending on the infusion method used during the CCK-HIDA scan [4]. This raises concerns about the diagnostic specificity of the current criteria for HBD and emphasizes the need for standardization in diagnostic approaches. Among other diagnostic tests, normal and abnormal results often overlap, as seen in laboratory values and radiologic tests. Correlating these findings with clinical evaluation is crucial. Additionally, future studies may consider stratifying and reporting the percentage of the population with and without test abnormalities and associated symptoms to enhance diagnostic precision.

Our findings suggest that cholecystectomy is an appropriate consideration for managing HBD, particularly in patients with persistent biliary symptoms refractory to conservative management. In all studies meeting the inclusion criteria, the majority of patients were found to have improvement in symptoms following cholecystectomy. However, the degree of improvement and number of patients affected widely varied, and the outcomes of non-operative management were not reported, making direct comparisons difficult. This variability across studies likely stems from lack of standardized diagnostic criteria and treatment protocols given that HBD remains a relatively new diagnosis. Addressing these gaps will be essential to strengthen future evidence and refine clinical decision-making. Standardizing the criteria used to assess symptom resolution post-cholecystectomy could improve comparability across studies in the field.

A prospective, multicenter randomized controlled trial comparing surgical and non-operative management in patients with HBD is urgently needed to define the true clinical benefit of cholecystectomy in this population. Such a study should stratify patients based on symptom profiles, ejection fraction thresholds, and diagnostic imaging protocols, while employing validated outcome measures such as the GI Quality of Life Index or SF-36 to allow for meaningful longitudinal comparisons. Standardizing the diagnostic process is equally critical. Future studies must adopt a uniform approach to HIDA scan interpretation, including consistent CCK dosing, infusion duration, and timing of image acquisition, to reduce diagnostic variability and improve reproducibility across institutions. If fatty meal ingestion becomes a preferred or adjunctive method of gallbladder stimulation, efforts should also focus on protocolizing its use by defining caloric content, nutrient composition, timing, and patient preparation. Prospective comparisons between fatty meal- and CCK-stimulated HIDA scans, with correlation to post-cholecystectomy symptom improvement, will help determine the most physiologically appropriate and diagnostically reliable method. Until high-quality evidence is available, the consistency of findings from this systematic review and prior reviews supports cholecystectomy as a reasonable treatment option for managing HBD in appropriately selected patients.

## Conclusion

The results of this systematic review and meta-analysis propose that cholecystectomy may be an effective treatment option for patients diagnosed with hyperkinetic biliary dyskinesia (HBD), particularly those with persistent biliary symptoms refractory to conservative management. In the pooled analyses of 14 studies involving 416 patients diagnosed with HBD, the risk ratio for symptom relief post-cholecystectomy was 3.72 [95 % CI: 2.57–5.38], demonstrating a statistically significant benefit of surgical intervention. These results that are consistent with prior systematic review conducted in 2020, further supporting the potential role of cholecystectomy in managing this condition.

While these findings are promising, the current evidence base is limited by the retrospective nature of most studies, the absence of control groups and the lack of standardized diagnostic and outcome criteria. Future studies should prioritize prospective study designs, inclusion of control groups, and the use of validated quality-of-life instruments to assess outcomes. Additionally, longer follow-up periods and multi-center collaborations could enhance the robustness and generalizability of the evidence.

Until such data are available, cholecystectomy may be considered a reasonable therapeutic option for select patients with symptomatic HBD, particularly those with clear biliary symptoms refractory to conservative management. Nonetheless, developing a standardized diagnostic framework and conducting high-quality comparative studies remain critical to refining patient selection criteria and determining the long-term efficacy of surgical intervention in this emerging clinical entity.

## CRediT authorship contribution statement

**Duyen Quach:** Visualization, Project administration, Methodology, Investigation, Formal analysis, Data curation, Conceptualization, Writing – review & editing, Writing – original draft. **Kayla Nguyen:** Conceptualization, Writing – review & editing, Writing – original draft. **Gabriella Tavera:** Visualization, Writing – original draft. **Rachel Wright:** Visualization, Data curation, Conceptualization. **Zuhair Ali:** Supervision, Project administration, Conceptualization, Writing – review & editing, Writing – original draft. **Mike Liang:** Supervision, Project administration, Conceptualization, Writing – review & editing, Writing – original draft.

## Ethics approval

Institutional Review Board approval was not required for this study as it is a systematic review of publicly available data and does not involve human subjects research.

## Funding

This research did not receive any specific grant from funding agencies in the public, commercial, or not-for-profit sectors.

## Declaration of competing interest

The authors declare that they have no known competing financial interests or personal relationships that could have appeared to influence the work reported in this paper.
